# *Rickettsia amblyommatis* infecting ticks and exposure of domestic dogs to *Rickettsia* spp. in an Amazon-Cerrado transition region of northeastern Brazil

**DOI:** 10.1371/journal.pone.0179163

**Published:** 2017-06-08

**Authors:** Francisco B. Costa, Andréa P. da Costa, Jonas Moraes-Filho, Thiago F. Martins, Herbert S. Soares, Diego G. Ramirez, Ricardo A. Dias, Marcelo B. Labruna

**Affiliations:** 1 Department of Preventive Veterinary Medicine and Animal Health, Faculty of Veterinary Medicine, University of São Paulo, São Paulo, SP, Brazil; 2 Preventive Veterinary Medicine, Faculty of Veterinary Medicine, State University of Maranhão, São Luís, MA, Brazil; 3 Mestrado em Medicina e Bem estar animal, Universidade Santo Amaro, Av. Prof. Eneas de Siqueira Neto, São Paulo, Brazil; University of Minnesota, UNITED STATES

## Abstract

This study was performed in Maranhão state, a transition area two Brazilian biomes, Amazon and Cerrado. During 2011–2013, 1,560 domestic dogs were sampled for collection of serum blood samples and ticks in eight counties (3 within the Amazon and 5 within the Cerrado). A total of 959 ticks were collected on 150 dogs (9.6%). *Rhipicephalus sanguineus* sensu lato (s.l.) was the most abundant tick (68% of all collected specimens), followed by *Amblyomma cajennense* sensu lato (s.l.) (12.9%), *Amblyomma parvum* (9.2%), and *Amblyomma ovale* (5.2%). Other less abundant species (<1%) were *Amblyomma oblongoguttatum*, *Rhipicephalus microplus*, *Haemaphysalis juxtakochi*, and *Amblyomma rotundatum*. Females of *A*. *cajennense* s.l. ticks were morphologically identified as *A*. *cajennense* sensu stricto (s.s.) or *A*. *sculptum*. Molecular analyses of 779 canine ticks revealed three *Rickettsia* species: *Rickettsia amblyommatis* in 1% (1/100) *A*. *cajennense* s.l., ‘*Candidatus* Rickettsia andeanae’ in 20.7% (12/58) *A*. *parvum*, *Rickettsia bellii* in 6.8% (3/44) *A*. *ovale* and 100% (1/1) *A*. *rotundatum* ticks. An additional collection of *A*. *sculptum* from horses in a Cerrado area, and *A*. *cajennense* s.s. from pigs in an Amazon area revealed *R*. *amblyommatis* infecting only the *A*. *cajennense* s.s. ticks. Serological analysis of the 1,560 canine blood samples revealed 12.6% canine seroreactivity to *Rickettsia* spp., with the highest specific seroreactivity rate (10.2%) for *R*. *amblyommatis*. Endpoint titers to *R*. *amblyommatis* were significantly higher than those for the other *Rickettsia* antigens, suggesting that most of the seroreactive dogs were exposed to *R*. *amblyommatis-*infected ticks. Highest canine seroreactivity rates per locality (13.1–30.8%) were found in Amazon biome, where *A*. *cajennense* s.s. predominated. Lowest seroreactivity rates (1.9–6.5%) were found in Cerrado localities that were further from the Amazon, where *A*. *sculptum* predominated. Multivariate analyses revealed that canine seroreactivity to *Rickettsia* spp. or *R*. *amblyommatis* was statistically associated with rural dogs, exposed to *Amblyomma* ticks.

## Introduction

Amazon and Cerrado are the two largest biomes representing the greatest biodiversity of Brazil [1]. A few studies have reported the diversity of ticks infesting domestic dogs in areas within the Amazon [2–4] and Cerrado [5–7]. Overall, these studies indicate that dogs living in urban areas of counties within these two biomes are infested mainly by *Rhipicephalus sanguineus* sensu lato (s.l.), whereas dogs from rural areas are infested mainly by ticks of the genus *Amblyomma*, and also by *R*. *sanguineus* s.l. in some areas. While a number of *Amblyomma* species were reported on these dogs, *Amblyomma cajennense* sensu lato (s.l.) prevailed in the Cerrado biome, and only rarely reported on dogs from the Amazon biome.

The taxon *A*. *cajennense* s.l. was recently reassessed and demonstrated to represent a complex of six species, namely *Amblyomma cajennense* sensu stricto (s.s.) (restricted to the Amazonian region), *Amblyomma mixtum* (from Texas to western Ecuador), *Amblyomma sculptum* (northern Argentina, Bolivia, Paraguay, Brazil), *Amblyomma interandinum* (inter-Andean valley of Peru), *Amblyomma tonelliae* (dry areas of northern Argentina, Bolivia and Paraguay), and *Amblyomma patinoi* (Eastern Andes of Colombia) [8]. A subsequent extensive study demonstrated that only two of these species, *A*. *cajennense* s.s. and *A*. *sculptum* occur in Brazil [9]. In this case, the distribution of *A*. *cajennense* s.s. was primarily associated with the Amazon biome, whereas *A*. *sculptum* was primarily associated with the Cerrado and Atlantic rainforest biomes [9]. Interestingly, some transition areas were found with the two tick species in sympatry. One of these transition areas is the state of Maranhão, composed by the Amazon biome in the western part (where *A*. *cajennense* s.s. predominates), and by the Cerrado biome in the eastern and southern parts (where *A*. *sculptum* seems to predominate [9].

The *A*. *cajennense* species complex is of great medical importance in Brazil because *A*. *sculptum* is the main vector of *Rickettsia rickettsii*, the etiological agent of Brazilian spotted fever (BSF), the deadliest spotted fever of the world [10]. In southeastern Brazil, where BSF is endemic, *R*. *rickettsii* is the sole *Rickettsia* species that has been reported to infect *A*. *sculptum*; however, infection rates are extremely low among *A*. *sculptum* populations, usually with less than 1% of the ticks harboring *R*. *rickettsii* [11,12], albeit most of the *A*. *sculptum* populations are not found infected by any *Rickettsia* species [13–15]. On the other hand, the species *A*. *cajennense* s.s. is commonly found infected by another spotted fever group (SFG) agent, the species *Rickettsia amblyommatis* (formerly ‘*Candidatus* R. amblyommii’). Studies from the Brazilian Amazon have reported 26.8 to 69.4% *R*. *amblyommatis*-infection rates among *A*. *cajennense* s.s. populations [16,17]. While *A*. *cajennense* s.s. is an important human-biting tick in the Amazon region [9], this rickettsial agent was never confirmed as a human pathogen [18]), although there has been serological evidence that *R*. *amblyommatis* could cause spotted fever illness in the United States [19,20]. Importantly to note, there has been laboratory evidence that *R*. *amblyommatis-* naturally infected ticks are competent to transmit this rickettsial agent to rabbits [21].

Considering that the domestic dog is an important sentinel for tick-borne spotted fever [13,22,23], herein we investigated ticks infesting dogs and the rickettsial infection in ticks, and canine exposure to *Rickettsia* spp. in the state of Maranhão, where *A*. *sculptum* and *A*. *cajennense* s.s. have been reported to occur in sympatry in many areas.

## Materials and methods

### Study areas and canine sampling

This study was carried out in eight counties of the Maranhão state, northeastern Brazil. Three of the counties are located in the Amazon biome, whereas the remaining 5 counties belong to the Cerrado biome. Overall, the climate in the state of Maranhão is characterized by a rainy season extending from December to June, and the dry season from July to November. The annual average temperature, relative humidity and rainfall are 26.2°C, 70–85% and 1,000 to 2,500 mm, respectively [24].

From 2011 to 2013, a total of 1,560 domestic dogs were sampled. This sample included 160 to 260 dogs from each county, where most the times a similar number of urban or rural dogs was sampled (Table 1). Dogs from rural areas were sampled distantly at least 10 km from the urban area of each county. The minimum number of sampled dogs per county was previously calculated to be representative of each county, considering 18.9% estimate prevalence for rickettsial seropositivity [25], 6% error probability, and 95% confidence level, using Epitools epidemiological calculators (http://epitools.ausvet.com.au/). For this purpose, the canine population was estimated as 10% of the human population to each county. Every sampled dog was owned and was apparently health (with no external signs of clinical diseases) at the moment of sample collections. Canine health assessment was carried out by at least two veterinarians (FBC and APdaC) during sampling of every dog. The two veterinarians certified that dogs had normal behavior (no prostration, no locomotor or nervous disorder) and did not present any external sign of clinical disease (e.g., cutaneous diseases, fracture). If a dog had any sign of disease, it was not included in the present study.

**Table 1:** Ticks collected on dogs living in urban and rural areas of 8 counties from the state of Maranhão, northeastern Brazil.

Tick species	Counties *^a^*	Number of infested dogs (%) *^b^*		
		Urban: 737 examined dogs	Rural: 823 examined dogs	Total: 1,560 examined dogs
*Rhipicephalus sanguineus* s.l.	1–8	35 (4.7)a	66 (8.0)b	101 (6.4)
*Amblyomma cajennense* s.l.	1–3,5,7,8	2 (0.3)a	22 (2.7)b	24 (1.5)
*Amblyomma parvum*	4–6,8	0 (0.0)a	27 (3.3)b	27 (1.7)
*Amblyomma ovale*	1–3,5,8	10 (1.3)a	14 (1.7)a	24 (1.5)
*Amblyomma oblongoguttatum*	3,4	0 (0.0)a	3 (0.4)a	3 (0.2)
*Rhipicephalus microplus*	3,8	1 (0.1)a	1 (0.1)a	2 (0.1)
*Haemaphysalis juxtakochi*	6	0 (0.0)a	1 (0.1)a	1 (0.06)
*Amblyomma rotundatum* *^c^*	5	0 (0.0)a	1 (0.1)a	1 (0.06)
*Amblyomma* spp.	5,8	0 (0.0)a	3 (0.4)a	3 (0.2)

^*a*^ 1: Cururupu; 2: São Bento; 3: Açailândia; 4: Grajaú; 5: Barreirinhas; 6: Caxias; 7: São Domingos; 8: Balsas.

^*b*^ different letters in the same line means statistically differences of infestation rates.

^*c*^ this tick was found non-attached to the dog body.

### Ticks and hemolymph test

Each dog had its entire body examined for the presence of ticks, which were collected and placed into plastic vials containing a moistened piece of cotton to keep ticks alive until arriving at the laboratory. Ticks (adults and nymphs) were identified to genus and species according to current literature [9,26,27]. Larvae of the genus *Amblyomma* were not identified to species level due to lack of literature support.

Adult ticks that arrived alive in the laboratory were submitted to the hemolymph test for screening of rickettsial infection, as described [28]. For this purpose, a tick leg was cut and a drop of hemolymph was spread on a glass slide, which was stained by Gimenez staining. The tick was immediately frozen at -80°C for further processing. The slide was examined under an optical microscope with 1000x magnification with oil immersion, which identifies organisms morphologically compatible with bacteria of the genus *Rickettsia*.

### Isolation of *Rickettsia* in cell culture

Some of the ticks tested by the hemolymph test were randomly selected to be processed by isolation of rickettsiae in cell culture by the shell vial technique, as previously described [29]. Briefly, ticks were surface disinfected for 10 min in iodine alcohol, followed by several washes in sterile water. Then each tick was macerated by using a sterile mortar and pestle in 600μL of brain heart infusion broth. Two shell vials containing confluent monolayers of Vero cells were each inoculated with 300μL of the tick homogenate, and then centrifuged for 1h at 700 *g*. Thereafter, monolayers were washed and fed with 1 mL of minimal essential medium supplemented 10% with bovine calf serum and antibiotics [1% (each) penicillin, and streptomycin, and 1.5 g/ml amphotericin B), and incubated at 28°C. Every 3 days, the medium was switched to new medium without antibiotics, and the aspirated medium was checked by Giménez staining for the presence of *Rickettsia*-like organisms. If there were *Rickettsia-*like organisms, the monolayer of the shell vial was harvested and inoculated into a 25 cm^2^ flask containing a monolayer of confluent uninfected Vero cells. The level of infection of cells was monitored by Giménez staining of scraped cells from the inoculated monolayer. When ricketsial infection reached >90% of the cells, they were harvested and inoculated into 75-cm 2 flasks of Vero cells. The rickettsial isolate was considered to be established in the laboratory after at least three passages through 75 cm^2^ flasks, each achieving a proportion >90% of infected cells [29].

*Rickettsia-*infected cells of the third passage were submitted to DNA extraction by using the DNeasy Blood and Tissue kit (Qiagen, Chatsworth, CA). The extracted DNA was tested by polymerase chain reaction (PCR) protocols to amplify fragments of the rickettsial genes citrate synthase (*gltA*; primers CS-78, CS-323), the outer membrane proteins *ompA* (primers Rr190.70p, Rr190.701) and *ompB* (primers 190.59F, 190.807R), and the 17-kDa protein (*htrA*, primers 17k-5 and 17k-3), as described [29–31]. PCR products were purified using ExoSAP-IT (USB Corp., Cleveland, OH) and underwent DNA sequencing in an ABI automated sequencer (Applied Biosystems/Perkin Elmer, model ABI Prism 3500 Genetic, Foster City, CA), and the resultant sequences were compared with GenBank data by BLAST analysis (http://blast.ncbi.nlm.nih.gov/Blast.cgi).

### Molecular analysis of ticks

Ticks were individually submitted to DNA extraction by the guanidine isothiocyanate technique, as described elsewhere [13], and tested by PCR targeting a 401-fragment (primers CS-78, CS-323) of the rickettsial *gltA* gene [29]. Ticks positive by this PCR assay were tested by a second PCR assay (primers Rr190.70p, Rr190.701) targeting a ≈632 fragment of the SFG rickettsial *ompA* gene [30]. For each PCR run, a negative control (water) and positive control (*Rickettsia parkeri* strain NOD) were included. PCR products were DNA sequenced and submitted to BLAST analysis as described above. Body remnants of the shell vial-processed tick specimens were also included in these molecular analyses.

### Serological analyses of dogs

From each sampled dog, a blood sample was collected by venipuncture; the serum was separated by centrifugation, and kept frozen at -20°C until tested. Canine sera were tested by immunofluorescence assay (IFA) as described elsewhere [32] using Vero cells infected with each of the following five *Rickettsia* species known to infect ticks in Brazil: *R*. *rickettsii* strain Taiaçu [33], *R*. *parkeri* strain At24 [34] *R*. *amblyommatis* strain Ac37[16], *R*. *rhipicephali* strain HJ5 [35], and *R*. *bellii* strain Mogi [33] as crude antigens. In each slide, a serum previously shown to be non-reactive (negative control) and a known reactive serum (positive control) from a previous study [36] were included. Slides were incubated with fluorescein isothiocyanate-labelled rabbit anti-dog IgG (Sigma, St Louis, MO, USA). Sera showing antibodies titers to a *Rickettsia* species at least fourfold higher than those observed for the other *Rickettsia* species were supposed to be homologous to the first *Rickettsia* species or to a very closely related genotype [36,37].

### Additional collection of ticks

With the purpose to obtain additional adult ticks to be processed for isolation of rickettsiae in cell culture, *Amblyomma cajennense* s.l. ticks were collect from horses in Balsas county (July 2011) and from pigs in Viana county (August 2013). Ticks that arrived alive in the laboratory were submitted to the shell vial technique for isolation of rickettsiae in Vero cells, as described above.

### Statistical analyses

The proportions of tick-infested dogs or *Rickettsia-*seroreactive dogs between urban and rural areas were compared by the chi-square test or Fisher exact test. Canine endpoint titers to the five *Rickettsia* species were compared by the non-parametric Mann-Whitney test. For each sampled dog, a questionnaire was given to the dog owner with the purpose of gaining information about independent variables that could be associated with seroreactivity to *Rickettsia* spp. The following independent variables related to dogs were subjected to univariate analysis: living area (urban or rural), age (≤ 1 or > 1 year old), sex, breed (pure or mongrel), hunting activity (yes or no), living place near to forest (yes or no), tick species (only *R*. *sanguineus* s.l. or other species). Frequencies of independent variables were compared according to four dependent variables: seroreactivity (titer ≥64) to any of the five *Rickettsia* antigens; seroreactivity (titer ≥64) to *R*. *amblyommatis*; seroreactivity (titer ≥512) to *R*. *amblyommatis*; or seroreactivity to *R*. *amblyommatis* with endpoint titers at least four-fold higher than the titers to the other four *Rickettsia* species. Variables with statistical association (*P*<0.20, chi-square test or Fisher exact test) were tested in the multivariate model by the stepwise forward method. Four models, one for each dependent variable, were created. The variables were included in the multivariate model if they displayed statistical significance of *P* < 0.05. All analyses were performed using SPSS for Windows version 17.

### Ethical statement

This work has been approved by the Ethic Committee in the Use of Animals of the Faculty of Veterinary Medicine of the University of Sao Paulo (project number 2263/2011).

## Results

### Characterization of the sampled dogs

A total of 1,560 dogs (737 from the urban areas and 823 from rural areas) were evaluated in this survey. Overall, 919 (58.9%) dogs were males with a proportion of 394 (42.9%) from urban areas and 525 (57.1%) from rural areas. A total of 641 (36.2%) dogs were females, 343 (53.5%) from urban areas and 298 (46.5%) for rural areas. Only 5.7% of the sampled dogs were ≤ 1 year old, and almost all sampled dogs (99.6%) were mongrel. Hunting activity was reported for 213 (13.7%) dogs, from which 32 (15.0%) lived in urban areas and 181 (85.0%) in rural areas.

### Ticks on dogs

A total of 959 ticks were collected on 150 dogs (9.6%), being 6.1% (45/737) and 12.8% (105/823) of the urban and rural dogs, respectively. Overall, significantly more rural dogs were infested by ticks than urban dogs (*P*< 0.05). However, considering each of the eight counties separately, only Açailândia and Caxias had significantly more infested dogs in the rural area; the remaining 6 counties had statistically similar (*P>* 0.05) proportions of infested dogs among urban and rural areas.

*Rhipicephalus sanguineus* s.l. was the most abundant tick, with 652 (68%) specimens (307 males, 252 females, 88 nymphs, 5 larvae), followed by *A*. *cajennense* s.l. [124 (12.9%) specimens (13 males, 12 females, 99 nymphs)], *Amblyomma parvum* [88 (9.2%) specimens (17 males, 68 females, 3 nymphs)], and *Amblyomma ovale* [50 (5.2%) specimens (14 males, 36 females)]. Other less abundant species were *Amblyomma oblongoguttatum* [9 (0.9%) specimens (2 males, 7 females)], *Rhipicephalus microplus* [5 (0.5%) females], and *Haemaphysalis juxtakochi* [1 (0.1%) nymph]. A total of 29 (3%) specimens were retained as *Amblyomma* spp. One (0.1%) specimen of *Amblyomma rotundatum* was non-attached to the dog body.

Regarding the *A*. *cajennense* s.l. ticks, currently, only the female stage can be morphologically identified with certainty to species level (i.e., *A*. *cajennense* s.s. or *A*. *sculptum*). Among the 124 specimens of *A*. *cajennense* s.l. collected from dogs, only 12 specimens were females. Eight of these specimens were identified as *A*. *cajennense* s.s. (1 from Açailândia, 3 from Barreirinhas, and 4 from São Bento), and 4 specimens were identified as *A*. *sculptum* (2 from Açailândia and 2 from Balsas).

Considering each tick species separately, the proportions of infested dogs in rural areas were significantly higher (*P*<0.05) than in urban areas for *R*. *sanguineus* s.l., *A*. *cajennense* s.l., and *A*. *parvum*. The remaining tick species were found in much smaller numbers, and did not show significantly different proportions between urban and rural dogs (Table 1). Among the 150 infested dogs, most of them (120 dogs) were found infested by a single tick species. Twenty-seven dogs had dual infestations (simultaneous infestations by 2 tick species), usually *R*. *sanguineus* s.l. with a second tick species; 24 of these dogs were rural, and only 3 were urban. Triple infestations were found on only 2 rural dogs, which were simultaneously infested by *R*. *sanguineus* s.l. and 2 *Amblyomma* species.

### Infection and isolation of *Rickettsia* from ticks

A total of 99 tick specimens belonging to the species *A*. *cajennense* s.l., *A*. *ovale*, or *A*. *parvum* were screened by the hemolymph test, which indicated *Rickettsia-*like structures in 17 (17.1%) ticks (3 *A*. *ovale* and 14 *A*. *parvum*). Four of these ticks (2 *A*. *ovale*, 2 *A*. *parvum*) were tested by the shell vial technique. In addition, we also tested 9 negative-hemolymph ticks (5 *A*. *ovale*, 3 *A*. *parvum*, 1 *A*. *cajennense* s.s) by the shell vial technique, considering that the hemolymph test is not 100% sensitive for detection of rickettsiae in ticks [29]. None rickettsial isolation was established in Vero cells from these ticks.

A total of 779 ticks collected from dogs, including remnants of the 13 specimens processed by the shell vial technique, were tested by PCR targeting a fragment of the rickettsial *gltA* gene. Rickettsial DNA was detected in 12 *A*. *parvum* adults, 3 *A*. *ovale* adults, 1 *A*. *rotundatum* adult, and 1 *A*. *cajennense* s.l. nymph. The 12 *A*. *parvum* and the single *A*. *cajennense* s.l. nymph also yielded rickettsial DNA by the *ompA* PCR. Analyses of the DNA sequences generated from these PCR products indicated infection with ‘*Candidatus* Rickettsia andeanae’ in *A*. *parvum*, *Rickettsia bellii* in *A*. *ovale* and *A*. *rotundatum*, and *R*. *amblyommatis* in *A*. *cajennense* s.l. (Table 2). From the body remnants of the 13 tick specimens that were processed by shell vial, only 2 specimens (1 *A*. *ovale*, 1 *A*. *parvum*, both positive by the hemolymph test) were shown by PCR to contain rickettsiae.

**Table 2:** Results of molecular analyses for rickettsial infection in ticks collected on domestic dogs in the state of Maranhão, northeastern Brazil.

Tick species	Rickettsia infection		
	No. infected/No. tested (%)	Closest GenBank identity according to *Rickettsia* gene (accession number)	Counties*^a^*
*R*. *sanguineus* s.l.	0/541 (0.0)		
*A*. *cajennense* s.l.	1/100 (1.0)	100% *R*. *amblyommatis*: *gltA* (CP012420), *ompA* (KM042860)	3
*A*. *parvum*	12/58 (20.7)	100% ‘*Ca*. R. andeanae’: *gltA* (GU169050), *ompA* (KF179352)	6
*A*. *ovale*	3/44 (6.8)	100% *R*. *bellii*: *gltA* (CP000087)	1
*A*. *oblongoguttatum*	0/2 (0.0)		
*R*. *microplus*	0/5 (0.0)		
*H*. *juxtakochi*	0/1 (0.0)		
*A*. *rotundatum*	1/1 (100)	100% *R*. *bellii*: *gltA* (CP000087)	5
*Amblyomma* spp.	0/27 (0.0)		
Total	17/779 (2.2)		1,3,5,6

^*a*^ Localities where *Rickettsia-*infected ticks were found: 1- Cururupu; 3- Açailândia; 5- Barreirinhas; 6- Caxias.

### Additional collection of ticks

A total of 41 *A*. *cajennense* s.l. adults (5 males, 36 females) were collected from horses in Balsas County, and 93 *A*. *cajennense* s.l. adults (68 males, 25 females) were collected from pigs in Viana County (in the southern border of São Bento County). All females from Balsas were identified as *A*. *sculptum*, whereas all females from Viana were identified as *A*. *cajennense* s.s. A total of 5 *A*. *sculptum* and 14 *A*. *cajennense* s.s. were processed for isolation of rickettsiae by the shell vial technique. Rickettsial isolates were obtained in Vero cell cultures from 2 *A*. *cajennense* s.s. ticks. DNA fragments of the *gltA*, *htrA*, *ompA* and *ompB* genes of the two isolates were 100% (350/350-bp), 100% (419/419-bp), 99.8% (586/587-bp) and 99.9% (816/817-bp), respectively, identical to corresponding fragments in the genome of *R*. *amblyommatis* from Brazil (CP012420). In addition, the *ompA* fragment was 100% identical to a *R*. *amblyommatis ompA* fragment from the western Brazilian Amazon (KM042860), similarly to the *ompA* fragments amplified from canine ticks in this study (Table 2). However, only one Rickettsial isolate was established in Vero cells for at least 3 passages, since the second isolate was lost due to contamination by extracellular bacteria. Body remnants of the 19 shell vial-inoculated ticks were submitted to DNA extraction and PCR targeting rickettsiae; 5 *A*. *cajennense* s.s. ticks (including the 2 specimens that yielded rickettsial isolation) yielded *gltA* and *ompA* amplicons, which generated DNA sequences 100% identical to the established isolate of *R*. *amblyommatis*.

DNA partial sequences generated in this study have been submitted to GenBank and received the following accession numbers: KY628365-KY628368 for *R*. *amblyommatis gltA*, *htrA*, *ompA* and *ompB*; KY628369-KY628370 for *‘Ca*. R. andeanae’ *gltA* and *ompA*; and KY628371 for *R*. *bellii gltA*.

### Canine seroprevalence for *Rickettsia* spp.

**Fig 1:**
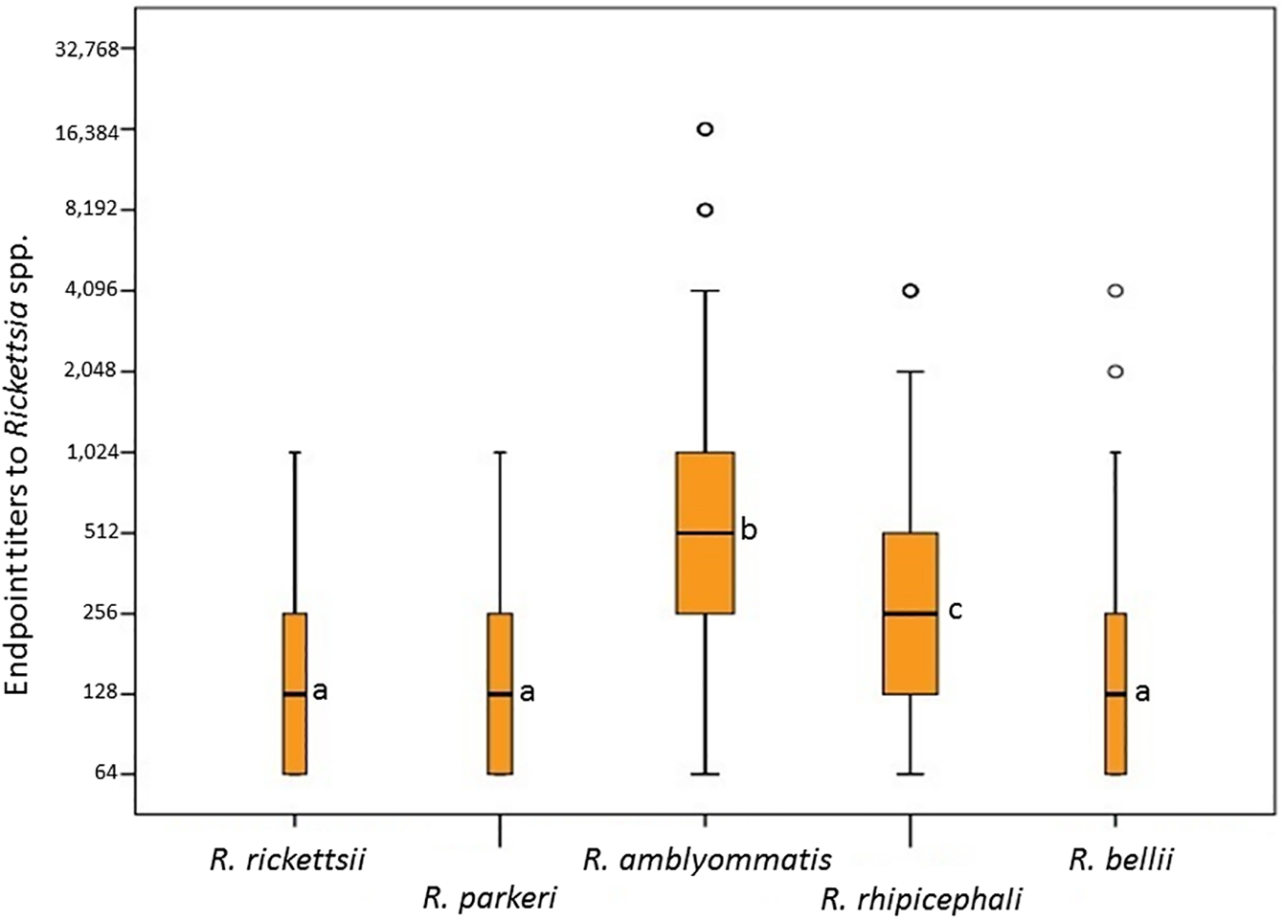
Boxplot representing the serological endpoint titers for five *Rickettsia* species of dogs from the state of Maranhão, an Amazon/Cerrado transition area in northeastern Brazil. Different lower case letters mean statistically different (*P*<0.05) endpoint titers between *Rickettsia* species.

A total of 1,560 canine serum samples were tested by IFA employing antigens of five *Rickettsia* species. Overall, 196 (12.6%) dogs were seroreactive to at least one *Rickettsia* species, with endpoint titers ranging from 64 to 16,384. The number of sera reacting at the screening dilution (1:64) to each *Rickettsia* species were 160 (10.2%) for *R*. *amblyommatis*, 151 (9.7%) for *R*. *rhipicephali*, 66 (4.2%) for *R*. *parkeri*, 64 (4.1%) for *R*. *rickettsii*, and 57 (3.6%) for *R*. *bellii* (Table 3). Endpoint titers to *R*. *amblyommatis* (median: 512) was significantly higher (*P*<0.05) than the endpoint titers to the other four *Rickettsia* species, for which the median values varied from 128 to 256 (Fig 1).

**Table 3:** Results of immunofluorescence assay (IFA) for five *Rickettsia* species in dogs from urban and rural areas of 8 counties in the state of Maranhão, northeastern Brazil.

County *^a^*	Area	No. seroreactive dogs to any *Rickettsia* species / No. tested dogs (% reactivity)	No. seroreactive dogs to each of *Rickettsia* species (% seroreactivity) *^b^*					No. of dogs with possible homologous reaction (PAIHR in parentheses) *^c^*
			Rr	Rp	Ra	Rrh	Rb	
Cururupu-A	Urban	14/105 (13.3)	3(2.8)	4(3.8)	12(11.4)	9(8.6)	5(4.8)	12 (10 Ra, 2 Rb)
	Rural	7/55 (12.7)	3(5.4)	3(5.4)	6(10.9)	6(10.9)	1(1.8)	
São Bento-A	Urban	19/121 (15.7)	9(7.4)	7(5.8)	19(15.7)	19(15.7)	9(7.4)	42 (38 Ra, 3 Rb, 1 Rrh)
	Rural	61/139 (43.9)	25(18.0)	27(19.4)	56(40.3)	55(39.5)	22(15.8)	
Açailândia-A	Urban	15/116 (12.9)	1(0.9)	1(0.9)	7(6.0)	4(3.4)	8(6.9)	12 (6 Ra, 6 Rb)
	Rural	15/44 (34.1)	5(11.3)	5(11.3)	10(22.7)	9(7.5)	6(13.6)	
Grajaú-C	Urban	8/100 (8.0)	2(2.0)	2(2.0)	6(6.0)	6(6.0)	0(0)	8 (6 Ra 2 Rrh)
	Rural	21/100 (21.0)	10(10.0)	8(8.0)	18(18.0)	20(20.0)	0(0)	
Barreirinhas-C	Urban	1/50 (2.0)	0(0)	0(0)	1(2)	0(0)	0(0)	1 (1 Ra)
	Rural	4/210 (1.9)	1(0.48)	1(0.48)	4(1.9)	2(0.95)	0(0)	
Caxias-C	Urban	0/59 (0.0)	0(0)	0(0)	0(0)	0(0)	0(0)	9 (9 Ra)
	Rural	9/101 (8.9)	2(1.9)	2(1.9)	9(8.9)	6(5.9)	1(0.9)	
S. Domingos—C	Urban	4/86 (4.6)	0(0)	4(4.6)	0(0)	0(0)	0(0)	3 (1 Ra, 1 Rb, 1 Rp)
	Rural	5/74 (6.8)	0(0)	0(0)	3(4)	4(5,4)	3(4)	
Balsas-C	Urban	0/100 (0.0)	0(0)	0(0)	0(0)	0(0)	0(0)	5 (2 Ra, 3 Rrh)
	Rural	13/100 (13.0)	3(3)	2(2)	9(9)	11(11)	0(0)	
Total		196/1,560 (12.6)	64(4.1)	66(4.2)	160(10.2)	151(9.7)	57(3.6)	92 (73 Ra, 12 Rb, 6 Rrh, 1 Rp)

^*a*^ County names followed by an “A” are within the Amazon biome; names followed by a “C” are in the Cerrado biome.

^*b*^ *Rickettsia* antigens: Rr: *R*. *rickettsii*; Rp: *R*. *parkeri*; Ra: *R*. *amblyommatis*; Rrh: *R*. *rhipicephali*; Rb: *R*. *bellii*

^*c*^ A homologous reaction was determined when an endpoint titer to a *Rickettsia* species was at least 4-fold higher than those observed for the other *Rickettsia* species. In this case, the *Rickettsia* species involved in the highest endpoint titer was considered the possible antigen involved in a homologous reaction (PAIHR).

A total of 73 (4.7%) canine sera from the eight counties showed endpoint titers to *R*. *amblyommatis* at least 4-fold higher than those to any of the other four antigens. The antibody titers in these 73 dogs were considered to have been stimulated by *R*. *amblyommatis* or a very closely related species (Table 3). Based on this criterion, 12 sera were considered to have been stimulated by *R*. *bellii*, 6 by *R*. *rhipicephali*, and 1 by *R*. *parkeri*. For the remaining 104 seroreactive dogs, it was not possible to discriminate the infection agent, because they displayed similar titers (< 4-fold difference) for two or more *Rickettsia* species or had a single titer of 64 for a single *Rickettsia* species.

**Fig 2:**
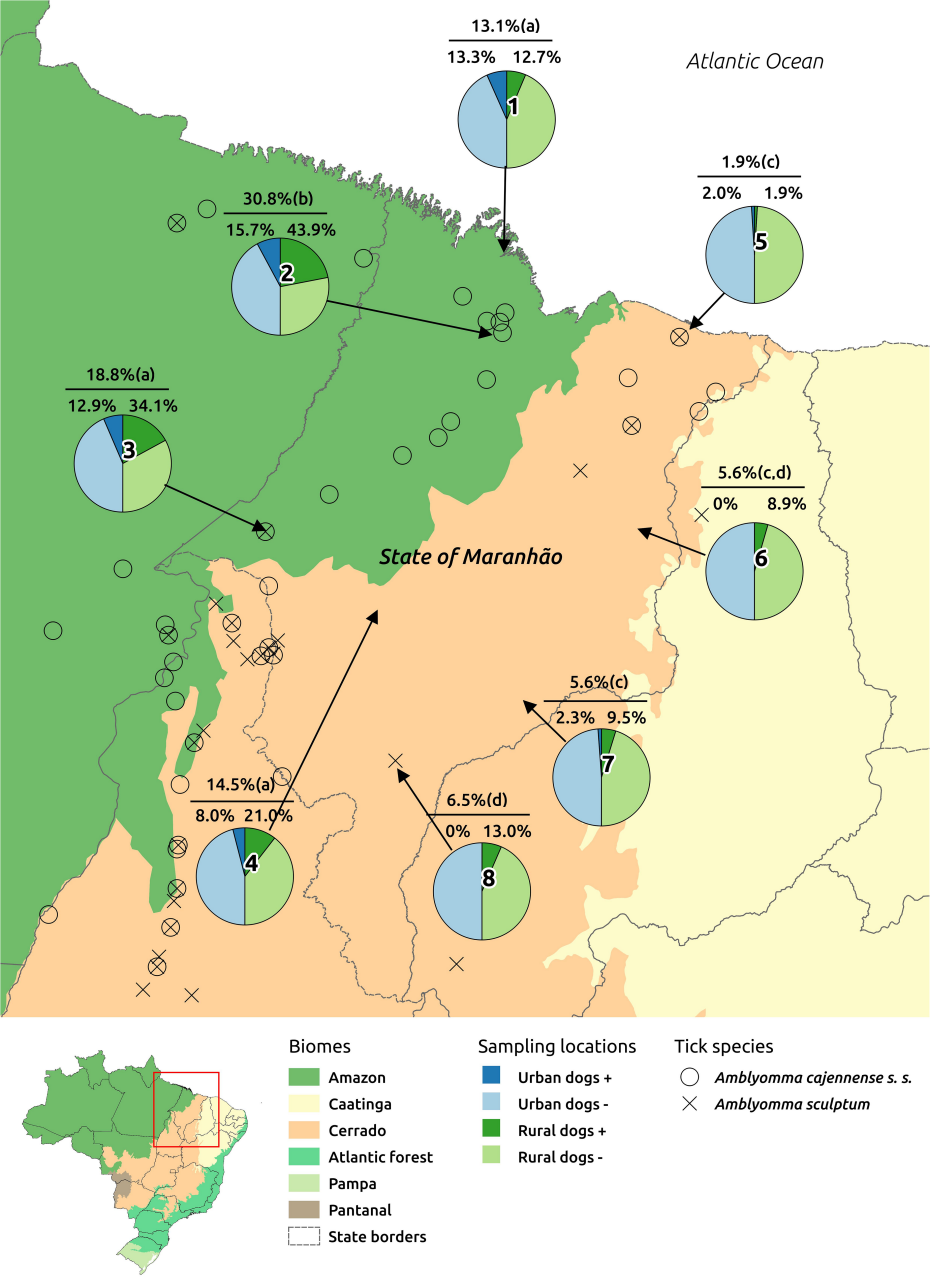
Localities (1- Cururupu; 2- São Bento; 3- Açailândia; 4- Grajaú; 5- Barreirinhas; 6- Caxias; 7- São Domingos; 8- Balsas) where domestic dogs were sampled in the state of Maranhão, northeastern Brazil. Pie charts represent the proportions of seropositive and seronegative dogs in urban and rural areas in each locality (Urban dogs +: seropositive dogs in the urban area; Urban dogs -: seronegative dogs in the urban area; Rural dogs +: seropositive dogs in the rural area; Rural dogs -: seronegative dogs in the rural area). Percent numbers above each pie chart represent canine seroreactivity values in urban and rural areas (lower numbers), and total canine seropositivity (top number). Different lower case letters inside parenthesis mean statistically different (*P*<0.05) total seroreactivity values between the 8 localities. The distribution of *Amblyomma cajennense* s.s. and *Amblyomma sculptum* in the map was retrieved from the study of Martins et al.[9].

In three counties (São Bento, Caxias, Balsas), rickettsial seroprevalence values were significantly higher (*P*<0.05) for rural than for urban dogs; in the remaining counties, seroprevalence values were similar (*P*>0.05) between urban and rural dogs. Considering the overall seroprevalence in each county, the highest seroprevalence value (30.8%, *P*<0.05) was found in São Bento in the Amazon biome, followed by Açailândia (18.8%) also in Amazon, Grajaú (14.5%) in Cerrado, and Cururupu (13.1%) in Amazon. The remaining four counties, all located in the Cerrado, had significantly lower (*P*<0.05) seroprevalence values, ranging from 1.9 to 6.5% (Fig 2).

By the univariate analysis, 4 canine independent variables (living area; age; hunting activity; proximity to forest; or tick species) showed statistical association (*P*<0.20) with each of the four dependent variables (S1 Table). These independent variables were selected for the multivariate analyses, which revealed that all four dependent variables related to canine seroreactivity to *Rickettsia* spp. or *R*. *amblyommatis* were statistically associated (*P*<0.05) with dogs living in the rural area, dogs with hunting activity, and dogs that were infested with ticks different from *R*. *sanguineus* s.l. (Table 4).

**Table 4:** Multivariate analyses by logistic regression for determination of risk factors (odds ratio) associated to four serologic profiles of domestic dogs tested by immunofluorescence assay (IFA)

Independent variables		Dependent variable: seroreactivity to *Rickettsia* spp. (titer ≥64)				
	Cases	Exposure	*P* value	Odds Ratio	CI (95%)	r^2^
Rural area	137	823	0.000	1.843	[1.314–2.586]	
Hunting activity	54	213	0.000	2.132	[1.465–3.104]	0.066
Ticks*	23	196	0.001	2.492	[1.433–4.334]	
		Dependent variable: seroreactivity to *R*. *amblyommatis* (titer ≥64)				
	Cases	Exposure	*P* value	Odds Ratio	CI (95%)	r^2^
Rural area	115	823	0.001	1.952	[1.338–2.848]	
Hunting activity	47	213	0.000	2.203	[1.476–3.290]	0.075
Ticks*	22	160	0.000	2.941	[1.670–5.180]	
		Dependent variable: seroreactivity to *R*. *amblyommatis* (titer ≥512)				
	Cases	Exposure	*P* value	Odds Ratio	CI (95%)	r^2^
Rural area	76	823	0.000	2.453	[1.492–4.034]	
Hunting activity	32	213	0.001	2.311	[1.437–3.717]	0.077
Ticks*	12	99	0.009	2.433	[1.246–4.751]	
		Dependent variable: seroreactivity to *R*. *amblyommatis* with titers 4-fold higher than the titers for other *Rickettsia* species				
	Cases	Exposure	*P* value	Odds Ratio	CI (95%)	r^2^
Rural area	53	823	0.020	1.946	[1.111–3.409]	
Hunting activity	25	213	0.000	2.606	[1.522–4.460]	0.068
Ticks*	11	73	0.010	2.576	[1.259–5.270]	

CI–Confidence interval

*Infestation by ticks different from *R*. *sanguineus* s.l.

## Discussion

A total of 8 tick species were collected from dogs in the state of Maranhão. This number excludes *A*. *rotundatum* because the collected specimen was not attached to dog, and includes two species of *A*. *cajennense* s.l. (*A*. *cajennense* s.s. and *A*. *sculptum*). Tick infestations were more frequent among dogs from rural areas, where there was also a greater diversity of tick species, when compared to dogs from urban areas. These findings are similar to previous studies that compared infestations between urban and rural dogs in Brazil [5–7]. On the other hand, our variety of 8 tick species infesting dogs is one of the greatest diversities found in Brazil, since previous studies encompassing rural dogs from all five major Brazilian biomes have reported 5 to 11 tick species for the Amazon biome [2–4], 3 to 7 tick species for the Cerrado biome [5–7,38,39], 2 to 6 tick species for the Atlantic rainforest biome [40–43], 2 tick species for the Pampa biome [44], and 1 tick species for the Caatinga biome [45].

In the present study, *Rhipicephalus sanguineus* s.l. was the most frequent tick among dogs from either urban or rural areas. Indeed, such finding is expected for urban areas, where human dwellings provide optimal conditions for the off-host development of *R*. *sanguineus* s.l. [2]. The predominance of *R sanguineus* s.l. among rural dogs also agrees with a number of previous studies from different parts of Brazil [5–7,40], although other studies reported higher prevalence of *Amblyomma* spp. ticks than *R*. *sanguineus* s.l. among rural dogs [39,42,43]. Such differences shall be related to canine habits; i.e., as more time dogs spend on human dwellings, higher the chances for establishment of *R*. *sanguineus* s.l. populations in rural areas. On the other hand, absence of *R*. *sanguineus* s.l. on rural dogs from some Amazonian sites [2,46] could be related to the yet non-introduction of this exotic tick into more remote areas, as discussed elsewhere [46]. Indeed, this is not the case of the state of Maranhão, where *R*. *sanguineus* seems to be widespread in rural areas.

Three rickettsial agents were detected in canine ticks: *R*. *amblyommatis* in an *A*. *cajennense* s.l. nymph, *‘Ca*. R. andeanae’ in *A*. *parvum*, and *R*. *bellii* in *A*. *ovale* and *A*. *rotundatum*. The *R*. *amblyommatis-*infected nymph is likely to belong to the species *A*. *cajennense* s.s., since this nymph was collected in Açailândia county (Amazon biome), where *A*. *cajennense* s.s. in known to occur. This statement is based on previous studies that reported that *R*. *amblyommatis* usually infects 26.8–69.4% of *A*. *cajennense* s.s. ticks [16,17], and at the same time, is usually not found in *A*. *sculptum* ticks [13–15]. In fact, our attempts to detect or isolate rickettsiae from *A*. *cajennense* s.s. and *A*. *sculptum* ticks collected from other domestic animals (pigs and horses) were successful only for *A*. *cajennense* s.s., from which *R*. *amblyommatis* was detected/isolated in 35.7% (5/14) of the ticks. Our findings of *‘Ca*. R. andeanae’ infecting 20.7% of the *A*. *parvum* ticks is concordant with previous studies from Brazil and Argentina, where *A*. *parvum* ticks were commonly found infected by this rickettsial agent [47,48]. Finally, the presence of *R*. *bellii* in *A*. *ovale* or *A*. *rotundatum* ticks has also been previously reported in Brazil [16,25,42,49]. No rickettsial infections were detected in any of 541 *R*. *sanguineus* s.l. attached to dogs. These findings contrast to studies in southeastern where a few *R*. *sanguineus* s.l. ticks collected from dogs were infected by the human pathogens *R*. *rickettsii* or *Rickettsia* sp. strain Atlantic rainforest [42,43,50]. Noteworthy, these other studies were from spotted fever-endemic areas where *R*. *rickettsii* or *Rickettsia* sp. strain Atlantic rainforest were primarily associated with canine ticks of the genus *Amblyomma*, what could have facilitated horizontal transmission between different tick species via dogs.

Overall, 196 (12.6%) dogs were seroreactive to at least one *Rickettsia* species, with the highest specific seroreactivity rate (10.2%) for *R*. *amblyommatis* (Table 3). Even though serological cross-reaction is very common between SFG *Rickettsia* species in dogs [36,51], 73 dogs (37.2% of all 196 seroreactive dogs) presented titers to *R*. *amblyommatis* at least four-fold higher than the titers to the other five *Rickettsia* antigens that were tested. Indeed, the overall endpoint titers to *R*. *amblyommatis* were significantly higher than those for the other *Rickettsia* species (Fig 1). These results suggest that most of seroreactive dogs of the present study have been exposed to *R*. *amblyommatis*-infected ticks. In fact, the *R*. *amblyommatis-*seroreactive dogs were statistically associated with infestation by ticks different from *R*. *sanguineus* (Table 4); e.g., *A*. *cajennense* s.l. ticks, the most frequent *Amblyomma* ticks, which were found infected by *R*. *amblyommatis*. In addition, considering the 8 sites of the present study, the highest canine seroreactivity rates (13.1 to 30.8%) were found in the western part of Maranhão state, composed basically by the Amazon biome, and consequently, with the presence/predominance of *A*. *cajennense* s.s. (Fig 2), which is naturally found infected by *R*. *amblyommatis* ([16,17], present study). On the other hand, the lowest seroreactivity rates (1.9 to 6.5%) were found in the eastern part of Maranhão state (Cerrado biome), where *A*. *sculptum* predominates (Fig 2). All these above statements provide strong epidemiological evidence for the natural infection of dogs, via tick transmission, by *R*. *amblyommatis* in the state of Maranhão. This evidence is also corroborated by a laboratory study showing that *R*. *amblyommatis* is tick transmitted [21]. In addition, one study in the United States showed canine seroconversion to *R*. *amblyommatis* after natural exposure to *Amblyomma americanum* ticks, which are frequently found infected by this rickettsial agent under high infection rates; these seroconverted dogs also showed much higher endpoint titers to *R*. *amblyommatis* than to other two SFG agents [51].

The third most abundant tick species in the present study, *A*. *parvum*, is a relatively common canine tick in the Cerrado biome [38,52], including in the present study (Table 1, Fig 2), where ‘*Ca*. R. andeanae’ was detected in 20.7% of the *A*. *parvum* ticks. While there is a possibility that canine exposure to ‘*Ca*. R. andeanae’-infected ticks could have interfered in our serological results, this possibility is unlikely considering that one study demonstrated that ‘*Ca*. R. andeanae’ is not efficiently transmitted to the host skin during tick feeding [53]. Finally, our serological results also suggest that a few dogs could have been infected by *R*. *bellii*, *R*. *rhipicephali*, or *R*. *parkeri* (Table 3). These three rickettsial agents have been isolated from some of the tick species that were collected on dogs in the present study; e.g., *R*. *bellii* from *A*. *ovale* ([42,43], present study), *R*. *rhipicephali* from *H*. *juxtakochi* [35], and *R*. *parkeri-*like agent (strain Atlantic rainforest) from *A*. *ovale* [42,43]. Due to low number of dogs with highest endpoint titers to these 3 rickettsial agents in the present study, more epidemiological background is required to support canine exposure by these agents in Maranhão. Finally, while *A*. *sculptum* is a natural vector of *R*. *rickettsii* in southeastern Brazil [12,11], our results showed no evidence for the circulation of this pathogen among domestic dogs, even though they have been considered efficient sentinels for *R*. *rickettsii* [13,22,36].

This study evaluated ticks, rickettsial infection in ticks, and exposure of dogs to *Rickettsia* spp. in an Amazon/Cerrado transition area of Brazil. Our results showed that the dogs, especially from the rural areas, are exposed to a great diversity of tick species, some of them carrying *Rickettsia* organisms. Dogs living in the Amazon biome of Maranhão (western part of the state) are much more likely to be seropositive to *R*. *amblyommatis* than dogs living in the Cerrado biome (eastern part of the state). This scenario is indeed related to the distribution of *A*. *cajennense* s.l. ticks in Maranhão, represented by *A*. *cajennense* s.s. ticks (usually infected by *R*. *amblyommatis*) mostly in the Amazon, and *A*. *sculptum* (usually not infected by *R*. *amblyommatis*) mostly in the Cerrado. Since *R amblyommatis* is suspected to be a human pathogen, surveillance of human infection by this agent should be directed to Amazon areas. On the other hand, if BSF (caused by *R*. *rickettsii*) is to be detected in Maranhão state someday, it is more likely to occur in the eastern part (Cerrado), where the natural vector (*A*. *sculptum*) predominates and there would be less interference by another SFG agent such as *R*. *amblyommatis*. This statement is based on previous studies that have shown that ticks cannot simultaneously maintain separate species of *Rickettsia* by vertical transmission, as recently discussed [54].

## Supporting information

S1 Table Results of univariate analysis (Fisher's exact test) for the association between independent variables with the serological results of domestic dogs, analyzed through four serologic profiles (four dependent variables) determined by the immunofluorescence assay: (i) canine seroreactivity to *Rickettsia* spp. (titer ≥64); (ii) canine seroreactivity to *R*. *amblyommatis* (titer ≥64); (iii) canine seroreactivity to *R*. *amblyommatis* (titer ≥512); (iv) canine seroreactivity to *R*. *amblyommatis* with titers at least 4-fold higher than the titers for other *Rickettsia* species. (DOCX)
