# Supplementary material for: Rickettsia amblyommatis infecting ticks and exposure of domestic dogs to Rickettsia spp. in an Amazon-Cerrado transition region of northeastern Brazil
Source: PLoS One. 2017 Jun 8;12(6):e0179163. doi: 10.1371/journal.pone.0179163 (PMC5464615; doi:10.1371/journal.pone.0179163)
Supplement: S1 Table — (DOCX) [file pone.0179163.s001.docx]

S1 Table: Results of univariate analysis (Fisher's exact test) for the association between independent variables with the serological results of domestic dogs, analyzed through four serologic profiles (four dependent variables) determined by the immunofluorescence assay: (i) canine seroreactivity to *Rickettsia* spp. (titer ≥64); (ii) canine seroreactivity to *R. amblyommatis* (titer ≥64); (iii) canine seroreactivity to *R. amblyommatis* (titer ≥512); (iv) canine seroreactivity to *R. amblyommatis* with titers 4-fold higher than the titers for other *Rickettsia* species.

|  | | Seroreactivity to *Rickettsia* spp. (titer ≥64) | | |  | Seroreactivity to *R. amblyommatis* (titer ≥64) | | |  | Seroreactivity to *R. amblyommatis* (titer ≥512) | | |  | Seroreactivity to *R. amblyommatis* with titers 4x > titers for other *Rickettsia* species | | |
| --- | --- | --- | --- | --- | --- | --- | --- | --- | --- | --- | --- | --- | --- | --- | --- | --- |
| Independent variables | | +/E | -/E | *P* |  | +/E | -/E | *P* |  | +/E | -/E | *P* |  | +/E | -/E | *P* |
| Area | Urban | 59/737  137/823 | 678/737  686/823 | <0.01 |  | 45/737  115/823 | 692/737  708/823 | <0.01 |  | 23/737  76/823 | 714/737  747/823 | <0.01 |  | 19/737  54/823 | 718/737  769/823 | <0.01 |
|  | Rural |  |  |  |  |  |  |  |  |  |  |  |  |  |  |  |
| Sex | Male | 121/919  75/641 | 798/919  566/641 | >0.05 |  | 98/919  62/641 | 821/919  579/641 | >0.05 |  | 65/919  34/641 | 854/919  607/641 | >0.05 |  | 44/919  29/641 | 875/919  612/641 | >0.05 |
|  | Female |  |  |  |  |  |  |  |  |  |  |  |  |  |  |  |
| Age (years old) | <1 | 5/89  191/1471 | 84/89  1280/1471 | <0.05 |  | 3/89  157/1471 | 86/89  1314/1471 | <0.05 |  | 2/89  97/1471 | 87/89  1374/1471 | >0.05 |  | 1/89  72/1471 | 88/89  1399/1471 | >0.05 |
|  | ≥1 |  |  |  |  |  |  |  |  |  |  |  |  |  |  |  |
| Hunting activity | No | 142/1347  54/213 | 1205/1347  159/213 | <0.01 |  | 113/1347  47/213 | 1234/1347  166/213 | <0.01 |  | 67/1347  32/213 | 1280/1397  181/213 | <0.01 |  | 48/1347  25/213 | 1299/1347  188/213 | <0.01 |
|  | Yes |  |  |  |  |  |  |  |  |  |  |  |  |  |  |  |
| Proximity to forest | No | 44/555  152/1005 | 511/555  853/1005 | <0.01 |  | 32/555  128/1005 | 523/555  877/1005 | <0.01 |  | 17/555  82/1005 | 538/555  923/1005 | <0.01 |  | 14/555  59/1005 | 541/555  946/1005 | <0.05 |
|  | Yes |  |  |  |  |  |  |  |  |  |  |  |  |  |  |  |
| *R. sanguineus* s.l. | No | 182/1459  14/101 | 1275/1459  87/101 | >0.05 |  | 147/1459  13/101 | 1312/1459  88/101 | >0.05 |  | 91/1459  8/101 | 1368/1459  93/101 | >0.05 |  | 65/1459  8/101 | 1394/1459  93/101 | >0.05 |
|  | Yes |  |  |  |  |  |  |  |  |  |  |  |  |  |  |  |
| Ticks * | No | 173/1485  23/75 | 1312/1485  52/75 | <0.01 |  | 138/1485  22/75 | 1347/1485  53/75 | <0.01 |  | 87/1485  12/75 | 1398/1485  63/75 | <0.01 |  | 62/1485  11/75 | 1423/1485  64/75 | <0.01 |
|  | Yes |  |  |  |  |  |  |  |  |  |  |  |  |  |  |  |

+: number of seroreactive dogs; -: number of seronegative dogs; E: exposure (number of examined dogs)

*Infestation by ticks different from *R. sanguineus* s.l.
